# 476. Increased Depression and Anxiety Associated with COVID-19 among Recipients of HIV Healthcare Services at Sexual Health Clinic in NYC

**DOI:** 10.1093/ofid/ofad500.546

**Published:** 2023-11-27

**Authors:** Simian Huang, Jason Zucker, Delivette Castor, Caroline Carnevale, Elijah LaSota, Joshua Klein, Tae Yoon Kim, Daniela Quigee, Deborah Theodore, Peter Gordon, Alwyn Cohall, Kathrine Meyers, Magdalena E Sobieszczyk

**Affiliations:** Columbia University Irving Medical Center, New York, New York; Columbia University Irving Medical Center, New York, New York; Columbia University Medical Center, New York, New York; New York Presbyterian Hospital, New York, NY; Columbia University Medical Center, New York, New York; New York Presbyterian, New York, New York; Columbia University Irving Medical Center, New York, New York; Columbia University, Department of Medicine, Division of Infectious Diseases, New York, New York; Columbia University Irving Medical Center, New York, New York; Columbia University Irving Medical Center, New York, New York; Columbia University, NY, NY; Columbia University Medical Center, New York, New York; Division of Infectious Diseases, Department of Medicine, Vagelos College of Physicians and Surgeons, New York-Presbyterian Columbia University Irving Medical Center, New York, NY, USA, New York, New York

## Abstract

**Background:**

The COVID-19 epidemic disrupted routine healthcare services and affected mental health. Within Stick2PrEP, a suite of studies aimed at increasing pre-exposure prophylaxis (PrEP) uptake and healthcare engagement among men who have sex with men (MSM) and trans women (TW) in New York City, participants enrolled from November 2018 to February 2020 (Stick2PrEP2) and, after pandemic onset, from May 2021 onward (Stick2PrEP3). To understand the impact of the pandemic on the mental health of MSM/TW, we compared Stick2PrEP2 and Stick2PrEP3 participants' self-reports of depression and anxiety.

**Methods:**

All participants in the two studies completed a baseline enrollment questionnaire that surveyed demographics, sexual behaviors, depression, anxiety, and intimate partner violence. We compared baseline characteristics of Stick2PrEP3 vs Stick2PrEP3 among participants enrolled between November 2018 and April 2022 using relevant statistics (e.g., Pearson's Chi-squared test or Wilcoxon rank sum test).

**Results:**

We analyzed 345 participants, 141 in Stick2PrEP2 and 204 in Stick2PrEP3. Participants were similar in age, self-identified gender, race/ethnicity, income, rates of adverse childhood events, HIV and sexually transmitted infection (STI) risk scores, and PrEP adherence norms. More participants were on Medicaid or uninsured in Stick2PrEP3 (p< 0.001). Additionally, both PHQ9 (2 vs. 4, p< 0.001) and GAD7 (2 vs. 4, p=0.002) scores were significantly higher in Stick2PrEP3. More participants reported experience of intimate partner violence (24% vs. 45%, p=0.002) in Stick2PrEP3, but had higher PrEP engagement (INDEX score >45, 66% vs. 86%, p< 0.001).

**Figure:**
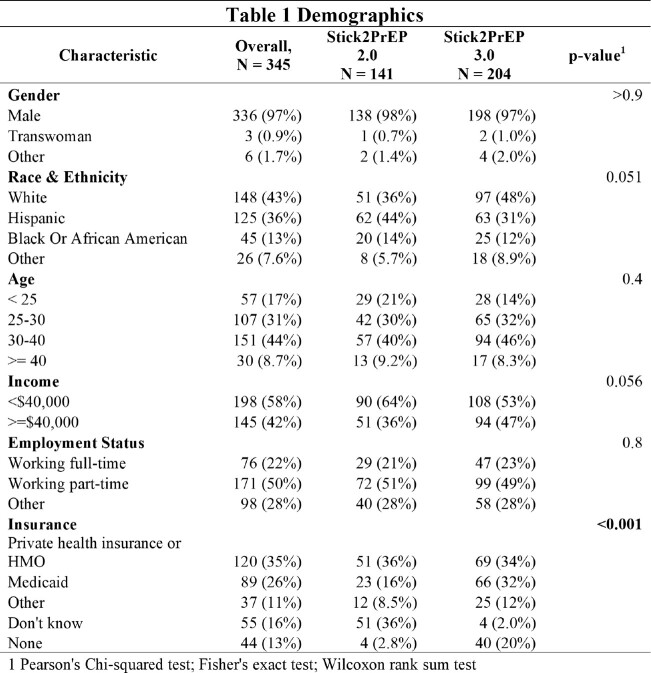

Demographic information of participants in Stick2PrEP2 and Stick2PrEP3.

**Figure:**
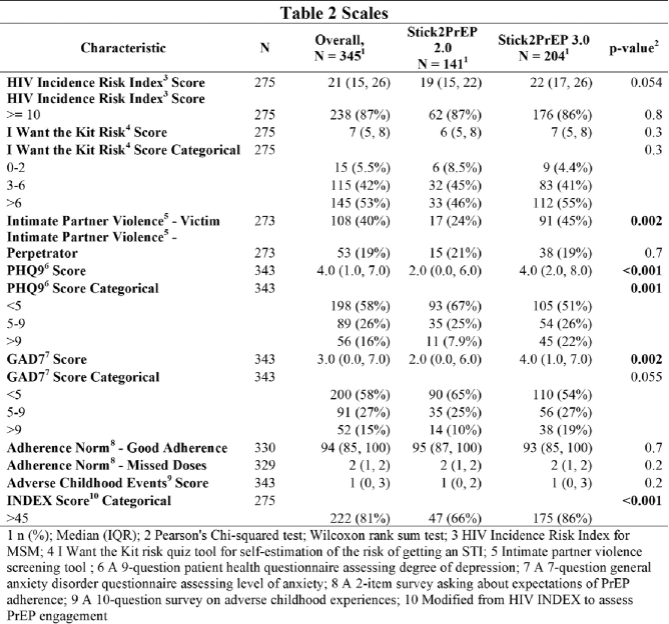

Results of different measurements from the enrollment survey that each participant completed.

**Conclusion:**

The baseline results suggest that depression and anxiety levels were lower before COVID-19 among MSM and TW. The experiences during the COVID-19 pandemic may have contributed to increased depression and anxiety levels which may be multifactorial and modified by disruption in PrEP services. Further investigation is crucial to uncover the link between COVID-19, mental health, and health services delivery for people at risk of HIV acquisition.

**Disclosures:**

**All Authors**: No reported disclosures

